# Effects of an intervention with teachers in the physical education context: A Self-Determination Theory approach

**DOI:** 10.1371/journal.pone.0189986

**Published:** 2017-12-28

**Authors:** David Sánchez-Oliva, Juan J. Pulido-González, Francisco M. Leo, Inmaculada González-Ponce, Tomás García-Calvo

**Affiliations:** 1 Faculty of Sport Science. University of Extremadura. Cáceres (Spain); 2 Faculty of Teaching Training. University of Extremadura. Cáceres (Spain); Kyoto University, JAPAN

## Abstract

Framed within Self-Determination Theory, the purpose of the present study was to test the effects of a training program with physical education (PE) teachers. Participants were 21 high school PE teachers (experimental group, *n* = 10; control group, *n* = 11), and their 836 students, aged 12 to 16 years. Teachers in the experimental group received a training program consisting of strategies to support autonomy, competence, and relatedness need satisfaction. A repeated measures ANCOVA was carried out for each dependent variable. After the intervention, students in the experimental group significantly increased their scores on autonomy support, relatedness support, autonomy satisfaction, autonomous motivation, controlled motivation, and intention to be physically active, as compared to the control group. These findings emphasize the utility of a training program with PE teachers to promote the students’ psychological need satisfaction, and hence, self-determined motivation toward PE classes.

## Introduction

During the past years, there has been a growing body of research aimed at determining students’ motivational processes during Physical Education (PE) classes, which has confirmed that students whose autonomy, competence, and relatedness are satisfied and who present a good quality of motivation are the ones who tend to develop more adaptive outcomes in PE classes, such as enjoyment, effort, vitality, positive affect, or the intention to be physically active in their free time [1–3], as well as higher levels of physical activity outside of school [4]. However, these studies suggest an interesting question: What elements of the learning environments that allow students to satisfy their needs of autonomy, competence, and relatedness and, therefore, present a good quality of motivation?

To answer this question, it is necessary to perform experimental studies in which intervention programs are carried out with teachers, in order to provide them with a set of motivational strategies that create a positive learning environment during PE classes [5]. Therefore, the purpose of this study was to examine the effects of an intervention with PE teachers, made up of methodological strategies to promote autonomy, competence, and relatedness satisfaction in students, in order to analyze the changes produced in perceived teacher’ motivational style and needs satisfaction, as well as the evolution of the type of motivational regulation and intention to be physically active after school.

## Self-determination theory

To address the motivational processes in students in PE classes, the present study draws from the Self-Determination Theory (SDT) [6,7]. SDT underlines the existence of three basic psychological needs (BPNs), autonomy, competence and relatedness, which are psychological nutrients for an optimal motivation. In the PE context, autonomy satisfaction emerges when students feel like the initiators of their own behavior and they participate voluntarily in the proposed activities. Competence satisfaction refers to a feeling of effectively interacting with the environment, developing feelings of achievement when performing tasks. Lastly, relatedness satisfaction refers to experiencing positive interaction with the rest of the classmates, developing feelings of belonging in the class context.

Cognitive Evaluation Theory [6], a mini-theory within SDT, focuses on the explanation of the social factors that can promote BPNs satisfaction. Within the PE context, the teaching environment plays a key role, emphasizing the teaching style as a basic component in the teaching-learning process. Traditionally, the teacher’ motivational style has been described using a continuum ranging from controlling (dark side) to autonomy-supportive style (bright side) [8]. Nevertheless, the bright side of the teacher’s interpersonal style has been extended to a tri-dimensional construct, providing support for the needs of autonomy, competence, and relatedness. Specifically, autonomy support refers to the teacher behaviors that take the student's perspective into account and allow freedom of expression and action of students, through the transfer of responsibility for decision-making when they perform tasks providing instruction to develop students' resources for autonomous self-regulation. Thus, an autonomy-supportive environment is characterized by attending to students’ interests and preferences, encouraging them to take control of their behavior, using informational and non-controlling language, and providing explanatory rationales [8]. Likewise, structure refer to the ability of a teacher to communicate information regularly to their students to guide their performance, to promote their sense of confidence and to achieve the aims proposed. A structured environment emerges when the social context is predictable, contingent, and consistent, and examples of structured behaviors are offering help during the tasks, giving enough time to achieve the goals, or promoting pupil learning and improvement in the activities [9,10]. Lastly, relatedness support strategies refer to the resources provided by the teacher to create learning environments that promote feelings of inclusion, integration, trust, and respect among the classmates. Being close, friendly, and empathic with the students, changing the strategy to form groups, or using specific activities (e.g., group dynamics) are examples of relatedness support behaviors [9,11].

Also, SDT considers people to be active organisms with innate tendencies toward growth and psychological development, distinguishing different types of motivation that vary according to one’s level of self-determination. Specifically, SDT propose that behavioral regulation can be divided into three large blocks, ranging from higher to lower self-determination: autonomous motivation, controlled motivation, and amotivation. Autonomous motivation is comprised of intrinsic motivation (students who are involved in the PE activities for the feelings of enjoyment, pleasure, interest, and satisfaction) and identified regulation (related to students who engage in an activity because they perceive the personal relevance of the PE activities). Controlled motivation is composed of introjected regulation (e.g., students who try to avoid guilty or shameful feelings when performing tasks) and external regulation (e.g., students who participate in activities to avoid punishment, obtain rewards, or meet external expectations, i.e., to get a better grade). Lastly, Amotivation is the lowest level of self-determination, associated with students who are not motivated either intrinsically or extrinsically and, therefore, they do not have intention of performing the activity [12,13]. Previous studies have shown that the students who perceive BPNs support by the teacher in PE classes show higher BPNs satisfaction, higher self-determined motivation, and higher positive outcomes, as enjoyment, subjective vitality, or physical activity intentions [1,10,14–18].

## Intervention programs with PE teachers

In recent years, various studies have assessed the effects of interventions focusing on the optimization of PE teachers’ interpersonal style [19]. For example, Chatzisarantis and Hagger [20] evaluated the utility of an intervention with 10 PE teachers on autonomy support strategies (by enhancing sense of choice and providing positive feedback, rationale and acknowledge difficulties within the PE classes) over a 5-week time interval. The results showed that the students from teachers receiving the intervention increased autonomy support, self-determined motivation, and self-reported leisure-time physical activity behavior when comparing to the control group. However, as acknowledged by the authors, the study did not include important variables such as needs satisfaction.

In a similar vein, Cheon et al. [21,22] carried out a research to test the effects of a training program to help PE teachers to be more autonomy supportive during instruction (through workshop-like experience, teaching scenarios, group discussion and hand-outs). They confirmed that, after the intervention program, students from the experimental group increased their levels of autonomy support, BPNs satisfaction, autonomous motivation, and future intention to practice physical activity, and they decreased their amotivation, in comparison with the control group. Cheon and Reeve [23] confirmed that the observed changes were maintained one year later, using the same participants in an extension of the previous study [21]. Likewise, Lonsdale et al. [24] examined the effects of an intervention program with PE teachers to develop autonomy support strategies, in which students in the intervention groups significantly improved their scores on the perception of choice provided by the teacher perception and autonomy satisfaction, but not on self-determined motivation, competence satisfaction, and relatedness satisfaction.

However, in our opinion, the above-mentioned studies have a common limitation due to the fact that the intervention programs focused exclusively on autonomy support strategies, thereby neglecting the development of strategies to promote competence and relatedness need satisfaction, and this fact also make it difficult to draw comparisons between studies. As established by Jang, Reeve, and Deci [10], autonomy support, structure and relatedness support are differentiated determinants that complement each other. Based on this information, we expect that a context where strategies of autonomy support, structure and relatedness support are provided may lead to more positive consequences compared to a context where only autonomy support strategies are provided.

Thus, a line of studies is emerging in which training programs with teachers are developed in order to provide the necessary strategies to create learning environments characterized by support of the three BPNs. Accordingly, Tessier, Sarrazin, and Ntoumanis [25] carried out a training program with three PE teachers, aimed at optimizing their teaching style (explaining to teachers the disadvantages of a reward–punishment system, and providing strategies to create the conditions under which students can motivates themselves), and analyzing the effects on students’ needs satisfaction, types of motivation, and engagement. For this purpose, after the initial assessment and systematic observation during four sessions, the teachers received training as a function of the results noted in the observation, using to improve autonomy support and structure strategies. After three classes, a final measurement of the students was performed, confirming that the program improved relatedness satisfaction and decreased non-self-determined motivation (external motivation and amotivation) significantly, but it did not produce improvement in autonomy satisfaction, competence satisfaction, and self-determined types of motivation. This study had two limitations, the participant teachers’ characteristics (newly qualified teachers) and the non-inclusion of a control group, which hinders generalization of the results. In a similar vein, Aelterman, Vansteenkiste, Van Den Berghe, De Meyer, and Haerens [26] carried out a study with 39 teachers and 669 students aimed to investigate the effects of a training program with PE teachers based on strategies to support autonomy (adopting an empathic attitude, providing choice, offering a rationale and integrating fun elements) and competence (giving an overview and communicating expectations, offering help, giving positive feedback, and encouragement). The intervention was effective to improve teachers’ beliefs regarding autonomy support and structure, as well as the perception of students and external observers regarding the use of autonomy support strategies (not in structure).

Taking into account the above-mentioned works, the present study provides an important contribution to the existing body of knowledge in two main ways. First, the current study develops a training program aimed at optimizing teachers’ interpersonal style, developing strategies to promote autonomy, competence, and relatedness need satisfaction (not only autonomy). Second, this study allows to tests the effects of the intervention on variables included at the four levels that make up the SDT [12]: perceived needs support (autonomy support, structure and relatedness support), needs satisfaction (autonomy, competence, and relatedness satisfaction), motivation (autonomous motivation, controlled motivation and amotivation), and outcomes (future intention to practice sport).

Thus, this study aimed to evaluate the effects of an intervention program in which PE teachers received support strategies of the three BPNs, to assess the changes in students’ motivational processes and different outcomes during PE classes. Specifically, the study hypotheses postulated that, compared to students in the control group, at post-test, students in the experimental group would show: (a) higher scores on perceived needs support and needs satisfaction; (b) higher scores on autonomous motivation and lower scores on controlled motivation and amotivation; and (c) an increase of intention to be physically active. Also, the fourth hypothesis of the study indicated that (d) there would be significant differences in the between-class variability, both intercept and slope.

## Method

Insofar as ethical rules are concerned, the study previously received the approval of the Ethics Committee of the University of Extremadura. All participants were treated in agreement with the ethical guidelines of the American Psychological Association with respect to consent, confidentiality and anonymity of the answers. Moreover, there was obtained an informed written consent from the parents on the behalf of the minors/children participants involved in the study.

### Participants

#### Participant teachers

The participant teachers were 21 PE teachers who taught in 21 public high schools of region of Extremadura (Spain). Teachers were aged between 30 and 49 years (*M* = 37.91, *SD* = 4.5), with teaching experience between 5 and 15 years (*M* = 10.95, *SD* = 4.62). The sample of teachers was performed randomly with the help of a purposeful selection, establishing the following requisites: (a) minimum experience of 5 years as a teacher; (b) having a minimum of two classes of both 1^st^ and 2^nd^ grades of Compulsory Secondary Education (CSE).

#### Participant students

Also, part of the sample were 836 students, ages ranging between 12 and 16 years (*M* = 12.81, *SD* = 0.93). Of them, 449 were enrolled in 1^st^ grade of CSE and 387 in 2^nd^ grade of CSE; regarding gender, 424 were male and 412 were female. Students belonged to 63 PE classes, with each class containing on average 13 students (range from 4 to 26 students). The 63 classes were grouped in 21 schools, with between 2 and 7 classes per school, and an average of 3 classes and 40 students per school. Participants who did not answer at least half of the measures were excluded from the study (21 cases; 2.42%). The questionnaires that showed unusual response patterns and response processes were also excluded from the study (12 cases, 1.38%).

### Measures

#### Need support

To assessed the autonomy support, structure and relatedness support, the Questionnaire of Basic Psychological Needs Support in Physical Education (CANPB) [27] was used. This instrument comprises the stem “*In Physical Education classes*, *my teacher*…”, followed by 12 items, four for each of the basic psychological needs support: autonomy support (e.g., “Often asks us about our preferences with respect to the activities we carry out”), structure (e.g., “Offers us activities based on our skill level”), and relatedness support (e.g., “Promotes good relationships between classmates at all times”). This scale was previously used within the Spanish PE context [16,28].

#### Need satisfaction

Autonomy, competence and relatedness need satisfaction were assessed using the Spanish adaptation for the context of physical education [29] of the Basic Psychological Needs in Exercise Scale (BPNES) [30]. Participants responded to the statement “In my PE classes…” by rating 12 items. Four items represented each of the basic psychological needs: autonomy satisfaction (e.g., “…we carry out exercises that are of interest to me”), competence satisfaction (e.g., “…I carry out the exercises effectively), and relatedness satisfaction (e.g., “…my relationship with my classmates is friendly”). The Spanish version of the BPNES has been used successfully in previous studies [31,32].

#### Motivation

To measure the different types of motivation, the Questionnaire of Motivation in Physical Education (CMEF) [33] was used. The questionnaire contained 20 items (4 items per behavioural regulation) that followed the statement “I take part in this PE class…”: intrinsic motivation (e.g., “Because PE is fun”), identified regulation (e.g., “Because I can learn skills that could be used in other areas of my life), introjected regulation (e.g., “Because I feel bad if I am not involved in the activities”), external regulation (e.g., “Because I want the teacher to think that I am a good student”) and amotivation (e.g., “But I think that I'm wasting my time with this subject”). In this study, autonomous motivation is composed by average of intrinsic motivation and identified regulation; and controlled motivation is comprised by average of introjected regulation and external regulation. This scale has been previously used within the Spanish PE context [16,28,34].

#### Intention to be physically active

One item was presented in the current study to measure students’ intention to practice sport in the following years: “In the following years, I have intention to practice sport”. Moreover, the questionnaire clarified that “practice sport” referred to the physical activity or sport practiced regularly during twice a week at least. Previous researches used a single-item satisfactorily [35,36].

Students respond to all items on a five-point scale ranging from *strongly disagree* (1), to *strongly agree* (5). All scales showed adequate internal reliability coefficients in this study (see Table 1).

**Table 1:** Descriptive statistics and internal reliability coefficients of the variables in pre-test and post-test.

	Total Sample (n = 836)		Control (n = 474)				Experimental (n = 362)			
	Pre	Post	Pre		Post		Pre		Post	
	*M(SD)*	*M(SD)*	*M(SD)*	*α*	*M(SD)*	*α*	*M(SD)*	*α*	*M(SD)*	*α*
Autonomy Support	3.90(0.90)	3.85(0.97)	4.07(0.82)	0.75	3.88(0.97)	0.82	3.68(0.95)	0.79	3.82(0.97)	0.82
Structure	4.50(0.64)	4.40(0.74)	4.53(0.65)	0.81	4.39(0.75)	0.86	4.48(0.62)	0.76	4.42(0.72)	0.81
Relatedness Support	4.39(0.69)	4.38(0.76)	4.46(0.67)	0.79	4.34(0.78)	0.84	4.31(0.72)	0.80	4.43(0.74)	0.82
Autonomy Satisfaction	3.77(0.93)	3.76(0.94)	3.90(0.93)	0.84	3.81(0.96)	0.84	3.60(0.91)	0.79	3.69(0.92)	0.81
Competence Satisfaction	4.12(0.78)	4.09(0.83)	4.16(0.79)	0.80	4.09(0.84)	0.83	4.07(0.77)	0.81	4.10(0.81)	0.81
Relatedness Satisfaction	4.38(0.71)	4.27(0.82)	4.41(0.69)	0.79	4.26(0.83)	0.87	4.34(0.72)	0.82	4.28(0.81)	0.85
Autonomous Motivation	4.23(0.73)	4.17(0.83)	4.27(0.74)	0.89	4.13(0.90)	0.92	4.18(0.72)	0.87	4.22(0.72)	0.88
Amotivation	2.24(1.29)	2.28(1.38)	2.34(1.31)	0.82	2.39(1.42)	0.89	2.10(1.26)	0.87	2.14(1.32)	0.89
Intention to be physically active	4.21(1.11)	4.19(1.11)	4.24(1.08)	-	4.15(1.10)	-	4.16(1.13)	-	4.23(1.10)	-

Standard Deviations are represented in the parentheses.

### Experimental design

The study was a 2 (Group; Control and Experimental) x 2 (Time; Pre-test and Post-test) randomized-experimental design. Teachers were randomly assigned to a control group and an experimental group. The control group was made up of 11 teachers and 474 students (225 females and 249 males; *M*_*age*_ = 12.97.81, *SD* = 0.96), and the experimental group included 10 teachers and 362 students (187 females and 175 males; *M*_*age*_ = 12.61, *SD* = 0.91).

At the beginning of January, all participants performed the pre-test, completing all questionnaires included in the study. Next, during the month of February, the teachers of the experimental group participated in the training program. After the formation, the PE teachers of the intervention group were asked to teach using the strategies of the training program. The intervention period consisted of 10 sessions, where all teachers were instructed to conduct a didactic unit planned for the “Games and Sport” content block (In the Spanish educational system, there are four blocks of contents: physical condition and health, games and sport, activities in nature and body language). During the intervention period, the first author remained in contact with the teachers to provide advice about strategies, answer any questions and ask about the development of the classes. All the teacher belonged to different centers, in order to avoid any influence in the teacher behaviors. Throughout the third trimester of the academic year (late April–mid May), all the students completed the questionnaires again, as the post-intervention measure.

### The intervention program with PE teachers

The teachers of the experimental group attended a training program based on the SDT postulates and the contributions of previously developed experimental studies [10,25,27], which was taught by two specialists in Sport Psychology and Educational Psychology. This training program lasted 15 hours, divided into three 5-hour sessions. It was carried out in a single room, and had a high level of practical components (i.e., videos, group dynamics, role playing, proposed practical or cases). Teachers always had opportunities to interact and ask any questions.

Part 1 lasted approximately 2 hours, where the main objective was to explain the theoretical background used in the study, that is, different types of motivational regulation (continuum of motivation), the importance of BPNs satisfaction to promote self-determined motivation, the influence of PE teacher' interpersonal style (supportive vs controlling style) on BPNs satisfaction and motivational regulation, and the incidence of motivational process on several outcomes on PE context (involvement, enjoyment, positive affect, intention to be physically active…). Part 2 lasted approximately 13 hours. Teachers were provided with several strategies to promote student’s autonomy, competence and relatedness satisfaction.

Strategies to be autonomy supportive focused on the importance of leadership and teaching style. Three important areas were developed: the importance of alternating teaching styles as a function of students’ needs; the need of giving freedom in students’ decision-making, and the advantages of avoiding controlling and pressuring behaviours. Later, strategies to promote active listening were implemented, such as promoting the students’ engagement, and using informational language when explaining task goals (e.g., avoiding”have to"). Finally, the teachers were also informed about the importance of increasing the responsibility of the students, in terms of taking the students’ perspective (the students’ interests, feelings, thoughts), and providing choice of some activities (e.g., the warm up) [8,10].

The main content for the strategies to promote competence was the adaptation of the learning according to the student's needs. To facilitate competence, focus centred on individualizing the content of the lessons with achievable challenges, achieving a balance between task difficulty and students’ skill, giving all the students the opportunity to achieve the goals, and allowing enough time to successfully complete the tasks. The relevance of directions and guidance were also presented, in terms of formulating clear, short-term goals, and rating success by means of intrapersonal indexes (assess the individual progress and not comparing progress to others). Lastly, other important strategies to support competence need is the use of an optimal feedback (before, during, and after the task), the use an adequate communication, and doing a private and meaningful assessment of the skills [10,37].

With the aim to promote relatedness need satisfaction in the students, teachers were recommended to adopt an empathic attitude. For example, being close, friendly and offering help to the students. Further, several methodological strategies were offered, such as variability in the strategy to form groups, optimizing the group’s control, and developing students’ social skills (empathy, active listening.). Finally, a set of specific activities were proposed in order to promote a good relationship of classmates, such as group dynamics, cooperative activities, role playing, activities to promote trust [37,38].

### Procedure

The main investigator contacted the schools to explain the goals of the study and request their participation. Prior to the data collection, all PE teachers were contacted and informed that the purpose of the study was to develop an experimental study designed to improve the students’ experiences and motivation during their PE lessons. All questionnaires were completed in class online via Google Doc Software, which is a software that allows users to create online surveys. Teachers explained the meaning of the items of the questionnaire to the students before they were completed to avoid any confusion. It was emphasized to the students that completion of the questionnaires was voluntary, their responses would be anonymous, and that they should answer honestly about their feelings toward PE. The questionnaires took approximately 25–30 minutes to complete.

### Data analysis

Data analysis consisted of two parts: preliminary analysis and intervention effects analysis. Initially, descriptive statistics of all dependent variables were calculated on pre-test and post-test, calculating the values of the total sample and according to the study group. Next, to examine the possible effect of Gender and Grade level, a one way Multivariate Analysis of Variance (MANOVA) on pre-test was performed.

Regarding intervention effects analysis, a mixed model with repeated measures ANCOVA was carried out for each of dependent variable of the study, including a between-subjects factor (Group), and three covariates (Time, Gender and Grade Level). The data were treated as a two-level model. Level 1 encompassed of repeated measures of each variable, and represents the change expected on each member of the population during the time period under study [39]. Level 2 consisted on between-class variance and represent the differences in growth rates between-class in random slope parameters. For each analysis, we have estimated 10 parameters. 6 fixed effects (Intercept, Group, Time, Group*Time, Gender, and Grade Level) and 3 random effects (Repeated measures variability, between-class intercept variability and between-class slope variability). Intercept refers to the estimation of the reference group (control group at pre-test), the Group effect estimated the difference between experimental group and control group at pre-test, the Time effect estimated the slope of the control group (difference between post-test and pre-test), whereas the Group*Time effect calculated the slope difference between experimental and control group (it is an indication of the effect of the intervention program). Regarding the random effects, the within-students variance refers to the students’ variability between measures. At the class level, the intercept variance indicates the between-class variability of the intercept, whereas the slope variance estimates the between-class variability of the slope [40]. Repeated measures were treated with Autoregressive Homogeneous (AR1) covariance structure, Restricted Maximum Likelihood (REML) was used as estimation method and random effect were analyzed with Diagonal covariance type and Wald test [40]. Effect sizes were calculated via odds ratio, using the following formula; ES=log(OR)=log(ai⋅dibi⋅ci), where a_i_ is the estimation of control group in pre-test; b_i_ is the estimation of control group in post-test; c_i_ is the estimation of experimental group in pre-test; and d_i_ is the estimation of experimental group in post-test. Cohen [41] proposed accepted criteria for the magnitude of the effect: <0.30 small effect size; 0.30–0.80 moderate effect size; >0.80 large effect size.

## Results

### Preliminary analysis

Descriptive statistics and internal reliability coefficients (Cronbach’s alpha) are presented in Table 1. All self-report measures showed acceptable levels of reliability, exceeding Nunnally’ criterion of 0.70 [42]. Also, we developed a validity analysis of the questionnaires. Using a first-order Confirmatory Factor Analysis (a three correlated-factors for need support and need satisfaction, and a five correlated-factors for motivation), all questionnaires showed a good fit to the data [43]: CFI > 0.90 and TLI > 0.90; RMSEA < 0.06 and SRMR < 0.08. All the factorial loadings of items on their specific factors were greater than 0.40 and statistically significant (p < 0.05).

Later, the possible associations between gender and grade level with dependent variables on pre-test were tested. MANOVA showed Gender was associated with all dependent variables, except structure, relatedness support and relatedness satisfaction. In all cases, boys reported higher scores (p < .01). Regarding effect of Grade Level, students of 1^st^ grade scored significantly higher on all dependent variables than students of 2^nd^ grade, except autonomy support, relatedness support, controlled motivation, and amotivation. Based on these results, Gender and Grade Level were included as covariates in subsequent analyzes.

### Intervention effects

Firstly, in order to examine possible between-class variation in the intercepts, we first tested a series of unconditional (intercept only) models, one for each variable under investigation, through the intraclass correlation coefficient [39]. This parameter is a quantification of the degree of between-class variability compared with the variability between-students of the same class, and therefore, it provides a sense of the degree of differences in the outcome between Level 2 units [44]. The results revealed that intraclass correlation coefficients scores ranged from 0.06 to 0.19 (*Mdn* = 0.12). Because the intercepts vary significantly across schools in all the dependent variables (3.52 < Wald Z < 4.63; p < 0.001), the development of a multilevel model is warranted [40].

#### Perceived needs support

**Fig 1:**
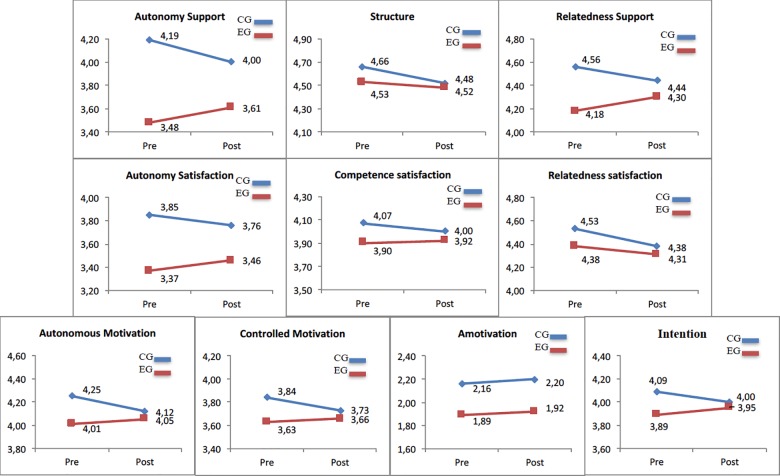
Scores for control group and experimental group on pre-test and post-test.

For autonomy support, at the beginning of the treatment the two groups were significantly different (p < 0.01). Intercept for the control group was 4.19, and experimental group was 3.48 (4.19–0.71). In terms of growth rates, control group revealed a negative growth (-0.19; p < 0.05) while students belonging to the experimental group reported significantly higher scores after the intervention time (0.13). There were significant differences in growth rates (p < 0.01), with a small effect size (ES = 0.08). For structure, the two conditions did not differ at baseline (control group = 4.66; experimental group = 4.66–0.13 = 4.53). Further, as illustrated by Fig 1, both groups had negative growth (control group = -0.14; experimental group = -0.05), with non-significant differences in growth rates (p > 0.05) and small effect size (ES = 0.02). For relatedness support, at the beginning of the treatment the two groups were significantly different (p < 0.01). Moreover, there were significant differences in the growths (p < 0.05), where control group had negative growth (-0.12), whereas experimental group grew at the rate 0.12, and a small effect size (ES = 0.05).

Regarding the random effects, the variability of level 1 was significant in the three needs support (p < 0.01). The intercept only varied between-classrooms at the beginning of the study in relatedness support (p < 0.05), while the slope varied between-classrooms in autonomy support (p < 0.01) and structure (p < 0.05).

#### Needs satisfaction

For autonomy satisfaction, as illustrated by Fig 1, at baseline the two groups were significantly different (p < 0.01). Intercept for the control group was 3.85 and for experimental group was 3.37 (3.85–0.48). In terms of slope, the group*time effect was significant (p < 0.05; ES = 0.08), where experimental group grew at the rate 0.09, while control group grew at a lower rate (-0.09). For competence satisfaction, the two condition did not differ at the beginning of the treatment (experimental group = 4.22; control group = 4.22 + 0.15 = 4.37). There were no significant differences in growth rates (p > 0.05; ES = 0.02), where experimental group grew at the rate 0.02 and control group grew at the rate -0.07. For relatedness satisfaction, at baseline the two groups were not different (p > 0.05), intercept for the control group was 4.53, and experimental group was 4.38 (4.53–0.15), whereas both groups had negative slope (control group = -0.15; experimental group = -0.07). There were no significant differences in growth rates (p > 0.05) and effect size was small (ES = 0.02).

In terms of random effects, the variability of level 1 was significant in the three needs satisfaction (p < 0.01). The intercept only varied between-classrooms at the beginning of the study on autonomy satisfaction (p < 0.05) while the slope varied between-classrooms on autonomy satisfaction, competence satisfaction, and relatedness satisfaction (p < 0.05).

#### Motivation

For autonomous motivation, as illustrated by Fig 1, at baseline the two groups were significantly different (control group = 4.25; experimental group = 4.25–0.24 = 4.01; p < 0.01). In terms of growth, control group had a negative growth at the rate -0.13, while experimental group grew at the rate 0.04, with significant differences in growth rates (p < 0.01) and small effect size (ES = 0.08). For controlled motivation, at the beginning of the treatment, there were no significant differences in intercepts (control group = 3.84; experimental group = 3.84–0.21 = 3.63; p > 0.05). In growth rates, it was significant differences (p < 0.05), with a small effect size (ES = 0.06), where control group grew at the rate -0.11 and experimental group grew at the rate 0.03. For amotivation, as illustrated Table 2, there were not significant differences in intercepts or slopes. Specifically, at baseline intercepts were not significantly different (control group = 2.16; experimental group = 1.89; p > 0.05). Further, both groups had positive growth (control group = 0.04; experimental group = 0.03), and it was not significant difference in growth rates (p > 0.05) with no effect size (ES = 0.00).

**Table 2:** Results of mixed repeated measures ANCOVAs in all variables.

		Fixed Effects Model						Within-Students		Between—Class	
	ICC	Intercept	Gender	Grade Level	Group	Time	Group * Time	Effect size	Intercept Variance	Intercept Variance	Slope Variance
Autonomy Support	0.14	4.19**	0.13*	.03	-0.71**	-0.19*	0.32**	0.08	0.74**	0.03	0.05**
Structure	0.11	4.66**	0.00	-.16**	-0.13	-0.14**	0.09	0.02	0.43**	0.01	0.01*
Relatedness Support	0.10	4.56**	0.00	-.04	-0.38**	-0.12**	0.24**	0.05	0.48**	0.04*	0.01
Autonomy Satisfaction	0.16	3.85**	0.29**	-.20*	-0.48**	-0.09	0.18*	0.05	0.72**	0.06*	0.03**
Competence Satisfaction	0.09	4.07**	0.29**	-.21**	-0.17	-0.07	0.09	0.02	0.58**	0.01	0.01*
Relatedness Satisfaction	0.10	4.53**	0.05	-.18**	-0.15	-0.15**	0.08	0.02	0.54**	0.01	0.02**
Autonomous Motivation	0.13	4.25**	0.29**	-.29**	-0.24*	-0.13**	0.17**	0.04	0.52**	0.01	0.02**
Controlled Motivation	0.14	3.84**	0.29**	-.14	-0.21	-0.11*	0.14*	0.06	0.61**	0.06*	0.02*
Amotivation	0.19	2.16**	0.37**	-.12	-0.27	0.04	-0.01	0.00	1.44**	0.23**	0.07**
Intention to be physically active	0.06	4.09**	0.44**	-.28**	-0.20	-0.09	0.15*	0.04	1.12**	0.04	0.00

**p < .01

*p < .05.

Gender (0 = Female; 1 = Male; reference category = Female); Grade Level (0 = 1° grade; 1 = 2° grade; reference category = 1° grade). Group (0 = control groups, 1 = Experimental group; reference category = Control group). Time (0 = Pre-test, 1 = Post-test; reference category = Pre-test).

Regarding the random effects, the variability of level 1 was significant on the three types of motivation (p < 0.01). The intercept varied between-classrooms at the beginning of the study on controlled motivation (p < 0.05) and amotivation (p < 0.01) while the slope varied between-classrooms on the three types of motivation (p < 0.01).

#### Intention to be physically active

For intention to be physically active, there were no significant differences in intercepts (control group = 4.09; experimental group = 3.89; p > 0.05). In terms of slope, it was significant differences (p < 0.05) and the effect size was small (ES = 0.04), where control group grew at the rate -0.09 and experimental group grew at the rate 0.06. Regarding the random effects, the variability of level 1 was significant (p < 0.01), the intercept did not vary between-classrooms at the beginning of the study (p > 0.05), while the slope varied between-classrooms (p < 0.05).

## Discussion

By means of the present work, we intended to develop a training program with teachers based on needs supportive strategies, with the aim of confirming the effects on the students’ motivational processes developed in the PE context and on future intention to practice physical activity. Data analysis was carried out by adjustment of the baseline scores, which permits deeper analysis of the utility of an intervention program designed for teachers to optimize the BPNs satisfaction, the type of student motivation, and adaptive consequences for the students during PE classes.

Specifically, the training program significantly improved both perceived autonomy support and autonomy satisfaction in the students from the experimental group, whereas the control students decreased their scores in both variables at post-test. That is, after the intervention, the students perceived that their teacher dedicated more resources to support their need for autonomy (e.g., adopting the students’ perspective, supporting students’ self-regulation, listening to students’ feeling and thoughts…) and, in addition, this increase in the strategies practiced by the teachers was reflected in an increase of students’ sense of control and, therefore, of their autonomy satisfaction. These results are consistent with other studies confirming that the intervention program produced a significant increase of autonomy support and autonomy satisfaction in the experimental group as compared with the control group [20,21,23,24]. These results are particularly relevant, because numerous studies have confirmed the great benefits for students when they improve their sense of volition, such as an improvement of enjoyment, effort, involvement during the PE classes, as well as a better attitude towards extracurricular exercise [19].

In contrast, the intervention program did not produce significant changes in structure or in competence satisfaction in the students from the experimental group as compared with the control students. In the case of structure, both groups decreased their scores after the intervention; that is, the training program was not sufficient to produce a change in students’ perception of the resources dedicated by the teacher to improve their competence satisfaction. The reason for these results may lie in the characteristics of the strategies aimed at improving competence satisfaction, (e.g., feedback, goals, directions, difficult level, type and duration of tasks…), which may be more difficult to perceive for students than autonomy and relatedness support strategies or, at short term, some of these strategies may even lead to the perception that their teacher is not helping them to improve their competence. Also, the estimation for the teachers belonging to the intervention group before the intervention was 4.47 (4.60 + (-0.13); the highest scores between all dependent variables), and it indicates that students belonging to the intervention group perceived their teachers as highly structured before the intervention, so these teachers had less room to improve through the training program.

Regarding the increase of competence satisfaction, the intervention program improved the scores of the students of the experimental group, whereas students of the control group decreased their scores, although the condition*time interaction was not significant. Prior studies did not achieve a positive effect in these variables [24,25]. This may be due to the short time interval between pre-test and post-test, which may not have been sufficient for the students to increase their perception of skill. This is ratified when taking into account the postulates and investigations on perceived competence from different theories [45,46], which establish that, in order to perceive a significant change in the perception of competence or skill in an activity, a certain amount of time must go by during which successful experiences are achieved that transform this perception. Likewise, the results obtained by Cheon et al. [21] and Cheon and Reeve [23] confirm this fact because, using a design made up of three measures, they significantly improved competence satisfaction. Perhaps a follow-up measure the end of the term could have confirmed significant changes in structure and competence satisfaction.

Lastly, the intervention program had a positive effect on relatedness support, but no improvements were observed in relatedness satisfaction. Thus, the intervention program changed the students’ perception of the strategies used by their teacher to promote relatedness. However, this was not sufficient to produce an increase in relatedness satisfaction, in contrast to the results found in prior studies [21,25]. The reason for our findings may lie in the characteristics of the Spanish educational system, where a large number of students have the same teacher and the same classmates for several academic terms. This may cause them to establish stable interpersonal relations and beliefs about the behavior of the teacher and their peers that are difficult to modify with a short intervention program.

Overall, these findings are in line with the postulates of SDT [6] and ratify the importance of the teacher’s interpersonal style, showing that the intervention program was effective to produce a change in students’ perception of the resources dedicated by the teacher to support autonomy and relatedness needs, and this led to an increase in autonomy satisfaction. However, the hypotheses were not confirmed in the case of structure, competence satisfaction, and relatedness satisfaction. As commented above, perhaps more time between the pre-test and post-test measures would have allowed the students to develop the set of strategies presented in the training program to a greater extent, which would then have produced a significant increase of competence and relatedness satisfaction at post-test.

With regard to motivational regulations, the results partially confirm our hypothesis. Autonomous motivation and controlled motivation increased similarly in both groups, although the students from the experimental group increased their scores significantly more than the control group after intervention period. Thus, the set of strategies practiced by the teachers increased the types of autonomous regulation (intrinsic and identified regulations). In view of the results obtained in BPNs satisfaction, this increase could derive from the increase in autonomy satisfaction, whereby the students developed a greater sense of control during PE classes, which could cause them to develop stronger practice motives that are intrinsic to the activity. These findings are consistent with tenets of self-determination [12] and they are similar to the results found in previous studies [20,21,23]. However, the results do not agree with the findings of Tessier et al. [25], in which no significant changes were found either in intrinsic motivation or in identified regulation.

In the case of controlled motivation, a priori, the findings do not fit the SDT postulates. However, according to Vansteenkiste, Niemiec, and Soenens [47], who explain motivation from a dual perspective that differentiates the quantity and quality of motivation, the results obtained could take an interesting turn. For instance, the training program improved both the quality (autonomous motivation) and the quantity of motivation (controlled motivation), leading to an increase in the motives for engagement in PE classes. Thus, as indicated by these authors, as long as the autonomous motivation levels increase, the increase observed in controlled motivation can have positive consequences. The specific characteristics of the context in which the investigation was carried out—where PE class attendance is obligatory, the teacher must grade the students publicly, and students must exceed a certain grade to pass the subject—should also be taken into account. These aspects can cause controlled motivation to increase as the assessment period approaches (as occurred in the chronology of this study), in spite of the teacher’s efforts to prevent this. To date, there are few studies that have tested the effects of an intervention program with teachers on students’ controlled motivation. Only Tessier et al. [25] reported that their training program did not produce notable changes in introjected regulation, but it did produce a significant decrease in external regulation.

With regard to amotivation, both groups increased their scores at post-test, and there were no significant differences in any of the cases. These results are not consistent with prior findings [21,23] indicating that students from the experimental group decreased their values significantly as compared with the control students. Similar results were obtained in boredom, in contrast to the hypothesis proposed. In the present study, these findings could be explained by means of random effects. Amotivation is the variable that obtained the greatest differences at the between-level. The fact that students participate in PE classes in a specific context (created by the teacher and the students) may partially explain their increased amotivation. In addition to the variables controlled for in the study, other factors related to the teacher and the classmates could have an impact on the evolution of amotivation during the term. Although a priori, some students may not increase their levels of amotivation, they might feel amotivated due to their perception of these feelings in their classmates.

Lastly, as hypothesized with regard to outcome, after the intervention, students from the experimental group significantly improved their scores in the intention to be physically active as compared with the control students. These results again ratify the benefits generated by the intervention program carried out with teachers, achieving improvements not only at the motivational level (BPNs satisfaction and type of motivation), but also in important attitudes toward physical activity outside school. The results are consistent with prior studies [21,23], and it confirms that motivational processes developed in PE classes play an essential role to promote out-of-school sport practice [4].

Regarding between-class level variability, the results showed significant differences in the intercept of relatedness support, autonomy satisfaction, controlled motivation, and amotivation, whereas slope variance had significant differences on all of dependent variables, except for relatedness support and intention to be physically active. That is, the learning environment created by the teacher and the students had little impact on the students’ scores at a specific point in time, but the results revealed the importance of the learning environment to produce a significant effect of the strategies practiced by the teachers. In general, these findings confirm the importance of the teacher’s figure, and the way teachers practice the strategies presented in the intervention program is fundamental.

Overall, these findings are in line with the postulates of SDT [6] and confirm the importance of the teacher’s interpersonal style, showing that the intervention program was effective at producing a change in students’ perceptions of the resources dedicated by the teacher to promote autonomy and relatedness needs, autonomous and controlled motivation, and intention to practice extracurricular sport. However, the hypotheses were not confirmed in the case of structure, needs satisfaction and amotivation. As commented above, perhaps more time between the pre-test and post-test measures would have allowed the students to develop the set of strategies presented in the training program to a greater extent, which would then have produced a significant increase in the non-significant variables in the post-test.

Among the limitations of the study, the main weakness was that we did not implement an observation of the teachers in order to determine the fidelity of the implementation. However, the large number of participants in the work and the vast geographical expanse of the region where the study was developed did not allow for a systematical follow-up of the strategies practiced by the teachers using an observational measure, like previous studies [26]. This aspect may be partially mitigated by the high representativeness of the participant’s sample and the continuous contact maintained with teachers during the intervention period. Also, the intervention program was limited to the supportive behaviors, and strategies to reduce need-thwarting behaviors were not implemented. This fact could explain (partly) the lack of effect on controlled motivation and amotivation. On the other hand, although the current study showed positive findings, they should be interpreted with caution, since effect sizes were small. Furthermore, the current study could be partially affected by Hawthorne effect [49], as the teachers of the control group did not have special attention. Another limitation of the current study is that, as we implemented a multi-component program, we could not estimate the specific effect of each teaching strategy on the dependent variables. An interesting research topic would be to carry out a study with four intervention groups (autonomy-supportive group, structured group, relatedness-supportive group, and three need-supportive group) in order to compare the potential effects in the experimental groups with separate trainings with those of the combined and control group. Furthermore, we are aware that the interval between the pre-test and the post-test could be insufficient to achieve all the proposed goals. Perhaps a follow-up at the end of the academic year would have encouraged the teachers to continue to use the strategies, and this would produce significant changes in other variables such as needs satisfaction.

## Conclusions

Ultimately, by means of the present study, we could confirm the positive effects of an intervention program with teachers on perceived need support and autonomous motivation within the PE classes. Furthermore, the results obtained ratify that PE context could be an interesting mean to promote future intention to practice sport after school [48]. In this regard, the public administrations should weigh the possibility of increasing the number of hours of PE and of training teachers in motivational aspects, with two goals: (a) to increase the time dedicated to sport practice at school; and (b) to improve students’ attitudes towards PE classes (self-determined motivation, enjoyment, satisfaction, importance of PE, positive affect, well-being…), which would lead to an increase of leisure-time sport practice [4] and thereby, to reducing future health expenditures to a large degree.
